# Crystal structure and substrate binding mode of ectonucleotide phosphodiesterase/pyrophosphatase-3 (NPP3)

**DOI:** 10.1038/s41598-018-28814-y

**Published:** 2018-07-18

**Authors:** Christoph Döhler, Matthias Zebisch, Norbert Sträter

**Affiliations:** 1 Institute of Bioanalytical Chemistry, Center for Biotechnology and Biomedicine, Leipzig University, Deutscher Platz 5, Leipzig, 04103 Germany; 2 Division of Structural Biology, Evotec, 114 Innovation Dr, Milton Park, Abingdon, Oxfordshire OX14 4RZ United Kingdom

## Abstract

Ectonucleotide phosphodiesterase/pyrophosphatase-3 (NPP3) is a membrane-bound glycoprotein that regulates extracellular levels of nucleotides. NPP3 is known to contribute to the immune response on basophils by hydrolyzing ATP and to regulate the glycosyltransferase activity in Neuro2a cells. Here, we report on crystal structures of the nuclease and phosphodiesterase domains of rat NPP3 in complex with different substrates, products and substrate analogs giving insight into details of the catalytic mechanism. Complex structures with a phosphate ion, the product AMP and the substrate analog AMPNPP provide a consistent picture of the coordination of the substrate in which one zinc ion activates the threonine nucleophile whereas the other zinc ion binds the phosphate group. Co-crystal structures with the dinucleotide substrates Ap4A and UDPGlcNAc reveal a binding pocket for the larger leaving groups of these substrates. The crystal structures as well as mutational and kinetic analysis demonstrate that the larger leaving groups interact only weakly with the enzyme such that the substrate affinity is dominated by the interactions of the first nucleoside group. For this moiety, the nucleobase is stacked between Y290 and F207 and polar interactions with the protein are only formed via water molecules thus explaining the limited nucleobase selectivity.

## Introduction

Ectonucleotide phosphodiesterase/pyrophosphatase-3 (NPP3, ENPP3, CD203c, PD-1β, gp130^RB13-6^) is a member of the NPP glycoprotein family, which comprises seven structurally related ectoenzymes (NPP1-7)^1–3^. All NPPs share a conserved zinc-binding catalytic domain (phosphodiesterase or PDE domain). Additionally, NPP1-3 consist of a nuclease-like domain and two N-terminal somatomedin B-like domains. NPPs hydrolyze pyrophosphate or phosphodiester bonds. Despite the structural similarity, the substrate specificity and the pathological and physiological function varies among the NPPs. NPP1, NPP3 and NPP4 hydrolyze nucleotides^4–6^, whereas NPP2, NPP6 and NPP7 dephosphorylate lipids^7–9^. NPP1 hydrolyzes ATP to AMP under release of pyrophosphate (PPi) and it is involved in bone mineralization and calcification in smooth muscle cells^5,10^. NPP2, also known as autotaxin, hydrolyzes lysophosphatidylcholine (LPC) to produce lysophosphatidic acid (LPA), which activates G-protein-coupled receptors inducing various cellular responses like cell growth and cell motility^11^. The hydrolysis of dinucleotides by NPP4 on the surface of the vascular endothelium stimulates platelet aggregation and secretion^6^. Due to a substitution inside the active center NPP5 is not able to cleave nucleotide triphosphates, but was found to convert adenine dinucleotide (NAD)^12^. The choline binding pocket of NPP6 allows the enzyme to hydrolyze glycerophosphocholine (GPC), a degradation product of phosphatidylcholine (PA), and to participate in the choline metabolism^13^. NPP7 has alkaline sphingomyelinase activity and controls cholesterol levels by converting sphingomyelin^14^. NPP3 is highly expressed in activated basophils and mast cells, rapidly induced by IgE and antigen-mediated crosslinking of the high-affinity IgE receptor FcεRI^15,16^. Therefore, NPP3 is used as an activation marker for clinical diagnosis of allergic diseases^17^. Recent studies showed that NPP3 negatively regulates chronic allergic responses by hydrolyzing extracellular ATP, which participates in the enhancement of allergic inflammation^18^. Hence, NPP3 could be a novel therapeutic target for allergic diseases. In Neuro2a cells NPP3 is suggested to have an intracellular function by modulating the level of intracellular nucleotide sugars during its transport from the endoplasmic reticulum through the Golgi lumen to the membrane^19^. The NPP3-mediated hydrolysis of UDP-N-acetylglucosamine (UDP-GlcNAc) produces UMP, which is a potent competitive inhibitor of N-acetylglucosaminyltransferase (GnT-IX). NPP3 has therefore been described as a key regulator for the function of GnT-IX and was shown to affect the cellular glycosylation profile.

NPP3 is also investigated as a target for anti-cancer therapy. The involvement of NPP3 in the control of differentiation and invasion could be shown for glia cells, although the molecular background of this process has not been clarified yet^20^. NPP3 was found to be expressed in several cancer cell types^21–25^, and is highly expressed in renal cell carcinoma, but has restricted expression in normal tissues, with the exception of the kidney^26^. Therefore, NPP3 is tested as cancer-specific antigen for antibody–drug conjugates in anti-tumor therapy^26^. Furthermore, NPP3 similar to NPP1 produces pyrophosphate from ATP in smooth muscle cells and could influence vascular calcification^5^. In receptive endometrial glands and stroma NPP3 is a progesterone regulated factor and could be used in a non-invasive test of endometrial receptivity in women^27^.

Crystal structures have been determined for NPP1^28,29^, NPP2^30^, NPP4^31^ NPP5^12^, NPP6^13^ and NPP7^32^. These structures revealed the conserved arrangement of the nuclease and PDE domains of NPP1 and NPP2 and the significant differences in the architecture of the substrate binding pockets for binding of nucleotide and phospholipid substrates while the core catalytic center comprising two zinc ions and an asparagine residue binding to the zinc-coordinated phosphate group is absolutely conserved. NPPs are related to alkaline phosphatase (AP) and the two enzyme families share the catalytic dizinc center with all metal ligands^3^.

In this extensive study, we determined the crystal structure of NPP3 from *Rattus norvegicus* in complex with a phosphate ion and various nucleotide ligands allowing us to characterize the substrate recognition of nucleotide triphosphates, dinucleotides and nucleotide sugars by NPP3. Analysis of the enzymatic activity with an ITC-based activity assay together with mutation experiments put the structural information in context. These data reveal a small selectivity of NPP3 for different types of nucleotides and the molecular background of this inselectivity. Furthermore, the recognition of larger substrates including the dinucleotide Ap4A is characterized by co-crystal structures and mutational analysis. Interestingly, although the various NPP3 ligand co-crystal structures reveal a similar catalytically competent substrate binding mode, a significant variation is seen in metal coordination in comparison with previously determined NPP complex structures.

## Results

### Crystal structure of NPP3

Crystals of NPP3 diffracted to 2.4 Å and contain two monomers in the asymmetric unit, which form a small interaction interface (536 Å^2^ as calculated by PISA^33^; Table 1). In size exclusion chromatography, the elution volume indicates the existence of monomeric protein (data not shown). In agreement with this finding, the crystal packing does not suggest the presence of a clear physiological dimer interface. In contrast, experiments on the membrane bound form demonstrated NPP3 dimers^34^. Thus, dimerization of NPP3 in the cell is most likely mediated by interactions of the transmembrane helices analogous to NPP1^29^. The two monomers in the asymmetric unit displayed no significant differences and superimposed with a root-mean-square-deviation (rmsd) of 0.165 Å.

**Table 1:** Data collection and refinement statistics.

	Apo NPP3	NPP3xAMP	NPP3^T206A^xAMPNPP	NPP3^T206A^xAp4A	NPP3^T206A^xUDPGlcNAc
PDB code	6F2T	6F2V	6F33	6F2Y	6F30
**Data collection**					
Beamline	BESSY MX 14.1	BESSY MX 14.1	BESSY MX 14.1	BESSY MX 14.1	BESSY MX 14.1
Wavelength (Å)	0.91843	0.91842	0.91841	0.91842	0.91800
Space group	P2_1_	P2_1_	P2_1_	P2_1_	P2_1_
Cell dimensions					
a, b, c (Å)	73.8, 124.1, 110.7	74.2, 125.3, 112.2	74.5, 126.9, 112.2	75.0, 126.8, 114.5	75.3, 127.7, 113.4
α, β, γ (°)	90, 97.0, 90	90, 97.0, 90	90, 96.8, 90	90, 97.5, 90	90, 97.1, 90
Resolution range (Å)	47.3–2.4 (2.45–2.4)	37.8–2.5 (2.54–2.5)	47.3–2.9 (2.95–2.9)	48.2–2.4 (2.45–2.4)	37.5–2.3 (2.34–2.3)
Completeness (%)	99.5 (100)	99.8 (99.8)	98.2 (98.7)	98.9 (99.8)	99.7 (99.9)
Multiplicity	3.4 (4.0)	3.6 (3.6)	2.8 (2.8)	3.0 (3.1)	3.4 (3.5)
〈*I*/σ(*I*)〉	7.6 (1.0)	12.3 (2.0)	2.4 (1.4)	7.8 (1.4)	9.9 (1.2)
*R*_r.i.m._(%)	13.1 (124.8)	9.7 (74.5)	23.7 (64.5)	12.2 (102.9)	8.7 (126.1)
CC_1/2_	99.5 (62.3)	99.7 (68.4)	95.7 (60.7)	99.5 (53.2)	99.8 (59.6)
Overal B factor from Wilson plot (Å^2^)	43.8	38.0	51.8	40.4	43.2
No. of Reflections	76975	70943	40761	81943	94004
**Refinement**					
Resolution range (Å)	47.4–2.4	37.8–2.5	47.3–2.9	48.2–2.4	37.5–2.3
*R*_work_/*R*_free_ (%)	21.5/22.9	19.4/20.9	24.5/26.8	20.4/21.7	19.6/21.3
No. of Atoms					
Protein	11524	11612	11597	11583	11610
Water	474	464	0	431	611
Glycans, Ligands, Ions	234	273	250	367	333
Average B-factors (Å^2^)					
Protein	55.6	50.0	68.2	53.6	56.4
Water	46.8	40.0	—	43.7	49.5
Ligands	70.0	62.7	73.9	71.5	75.4
R. m. s. deviation					
Bond length (Å)	0.007	0.007	0.007	0.007	0.007
Bond angles (°)	0.96	0.94	0.88	0.91	0.94
Ramachandran plot					
Favored region (%)	96.2	95.4	92.7	96.2	95.3
Allowed region (%)	3.6	4.5	7.1	3.6	4.5
Outlier region (%)	0.2	0.1	0.2	0.2	0.1

**Figure 1:**
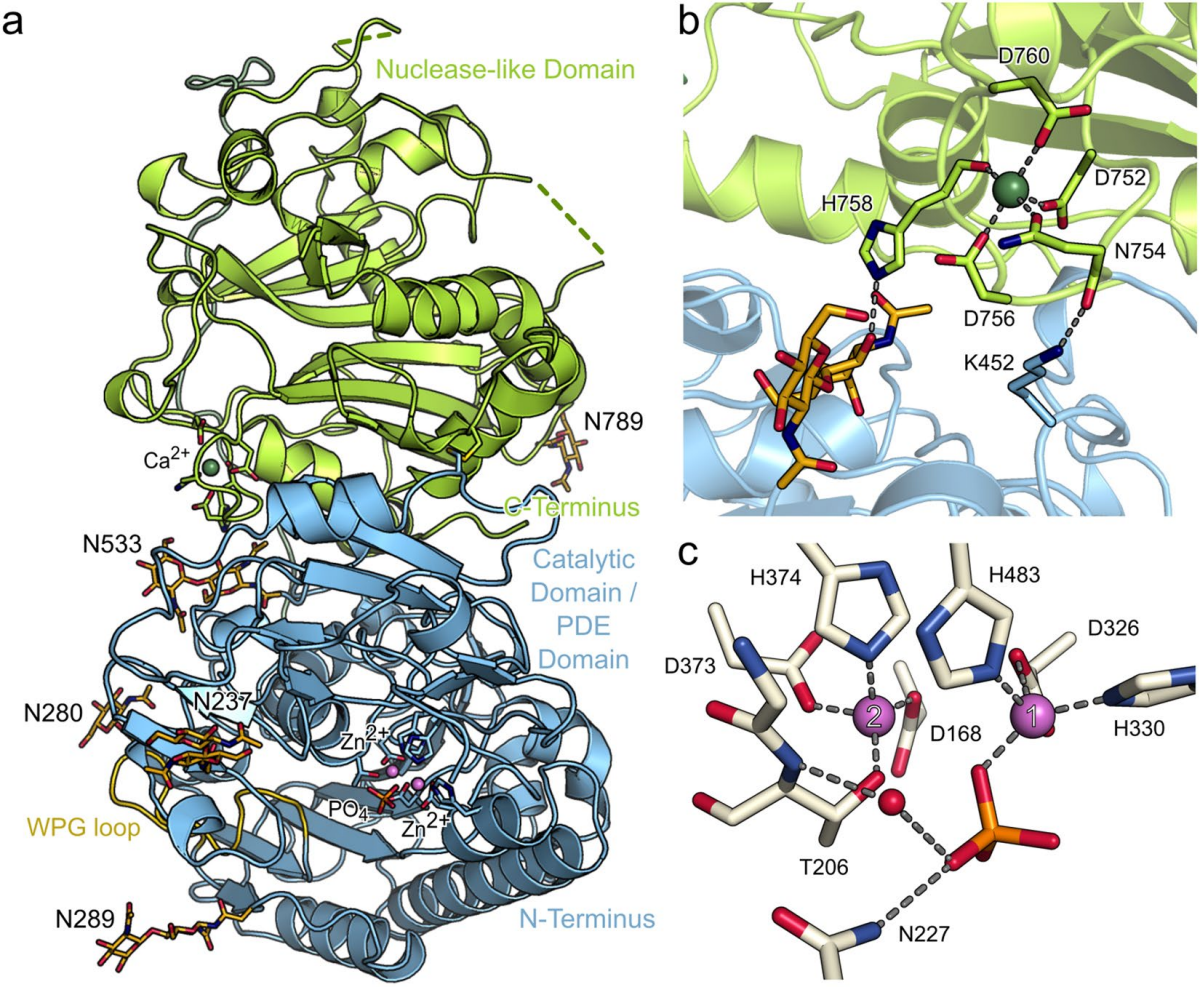
Crystal structure of NPP3. (**a**) Cartoon presentation of the domain organization of rat NPP3 with the catalytic domain/PDE domain depicted in blue and the nuclease-like domain in green. N-linked glycans are shown in stick representation, zinc and calcium ions as purple and green spheres, respectively. Residues complexing the metal ions and disulfide bridges are shown as sticks. (**b**) An EF-hand-like motif with a bound calcium ion interacts with the glycan chain at N533 close to the domain interface. (**c**) Coordination spheres of the two catalytic zinc ions and binding mode of a phosphate ion in the NPP3xPO_4_^3−^ structure.

Similar to NPP1 and NPP2, the structure of NPP3 consists of two major domains, which interact via a large interface of 1279 Å^2^ (Fig. 1a). Five salt bridges, 16 hydrogen bonding interactions and a loop of 18 residues (545–662) link the catalytic domain (residues 140–544) and the nuclease-like domain (residues 563–875). Additionally, a disulfide bridge between C429 and C818 stabilizes the domain arrangement. At the domain interface, a glycan chain at N533 of the PDE domain forms a hydrogen bond with H758 of the nuclease domain, which coordinates the Ca^2+^ ion of the EF-hand-like motif (Fig. 1b). This glycosylation site corresponds to N524 in NPP2 and is necessary for catalytic activity of this enzyme^35^. Additional glycosylation was clearly identified in the electron density maps at residues N237, N280, N289, N574, N702 and N789. For the predicted glycosylation site N594 no clear density was observed for monomer A in the asymmetric unit, which may be due to flexibility of this loop region. In monomer B, however, this region is well defined in the electron density maps due to crystal packing contacts. The electron density indicates that N594 is not glycosylated in monomer B.

In the nuclease-like domain, an EF-hand-like motif comprising residues D752, N754, D756 and D760 coordinates a Ca^2+^ ion (Fig. 1b). The Ca^2+^-binding site is also present in the structures of NPP1 and NPP2. This motif appears to be important for the structural integrity of the nuclease-like domain and so for the catalytic activity of NPP1^1^ and NPP3^36^ but not for NPP2^37^. In addition, N754 and K452 form a hydrogen bond interaction across the domain interface.

### Active center and substrate binding

The structure of the PDE domain of NPP3 is similar to the structures of the PDE domains of NPP1 (4GTW, 51% identity, root-mean-square-deviation (rmsd) of 0.75 Å), NPP2 (3NKM, 49% identity, rmsd of 0.88 Å), NPP4 (4LR2, 36% identity, rmsd of 1.45 Å), NPP5 (5VEM, 35%, rmsd of 1.32 Å), NPP6 (5EGE, 30% identity, rmsd of 1.54 Å) and NPP7 (5TCD, 30% identity, rmsd of 1.60 Å).

In the active site of NPP3 two Zn^2+^ ions (referred to as Zn1 and Zn2, Fig. 1c) are coordinated by D168, T206, D326, H330, D373, H374 and H483, which are strictly conserved in the NPP and alkaline phosphatase enzyme families. In addition, a phosphate ion is coordinated to Zn1 via one oxygen atom and it is also hydrogen bonded to T206. Zn1 exhibits a distorted five-fold coordination geometry, whereas Zn2 is coordinated tetrahedrally. The side chain of N227 is in hydrogen bonding distance to another phosphate oxygen, but the N-H···O angle of 113.4° indicates a very weak hydrogen bonding interaction.

To characterize the structural basis of substrate specificity of NPP3, we set out to determine co-crystal structures with products, substrates, and substrate analogs. In addition, the specificity for different substrates and the influence of active site residues for catalysis (Table 2) was investigated via kinetic analysis. To this end, we established a reaction assay based on isothermal tritration calorimetry (ITC)^38^. Using ITC the heat released during cleavage of the substrate high-energy phosphoanhydride bond can be detected without any labeling for many different substrates. The high sensitivity of an ITC experiment allows the determination of *K*_M_ in a low micromolar to a low millimolar range.

**Table 2:** Kinetic constants of the full length ectodomain of NPP3 and mutants determined for the hydrolysis of several substrates with an ITC-based enzyme assay.

Variant	Substrate	*K*_M_ [µM]	*k*_cat_ [s^−1^]	*A*_max_ [µmol min^−1^ mg^−1^]	*k*_cat_/*K*_M_ [M^−1^ s^−1^]
Wildtype	ATP	44 ± 2	10.0 ± 0.7	5.2 ± 0.4	226.8
	UTP	44 ± 3	11 ± 2	5.8 ± 1.0	255.1
	GTP	46.3 ± 0.5	3.2 ± 0.4	1.5 ± 0.4	70.3
	CTP	64 ± 17	10.8 ± 0.9	5.6 ± 0.5	169.6
	Ap3A	31 ± 2	18 ± 3	9.1 ± 1.5	565.9
	Ap4A	22.2 ± 0.1	18 ± 2	9.4 ± 0.8	812.0
	UDPGlc	133 ± 4	27.5 ± 0.7	14.3 ± 0.4	206.3
	UDPGlcNAc	176 ± 5	19 ± 3	10.0 ± 1.5	109.0
	NAD^+^	163 ± 10	17.5 ± 0.6	9.1 ± 0.3	107.7
K205A	ATP	54 ± 4	2.0 ± 0.7	1.0 ± 0.1	36.2
	Ap3A	124 ± 8	10.9 ± 0.1	5.6 ± 0.1	87.8
	Ap4A	109 ± 18	10.3 ± 1.2	5.4 ± 0.6	94.4
	UDPGlc	452 ± 59	31 ± 3	16.2 ± 1.5	68.7
	UDPGlcNAc	777 ± 105	20.1 ± 1.0	9.6 ± 1.5	25.9
T482V	ATP	46 ± 14	3.8 ± 0.2	1.9 ± 0.1	80.8
	UDPGlc	232 ± 5	14.0 ± 0.2	7.2 ± 0.1	60.1
	UDPGlcNAc	340 ± 43	9.9 ± 0.1	5.1 ± 0.5	29.0
G480A, G481A, G484A	Ap3A	94 ± 8	2.3 ± 0.1	1.2 ± 0.1	23.2
	Ap4A	37 ± 5	4.5 ± 1.2	2.3 ± 0.6	120.6
T379V	Ap3A	61 ± 16	2.9 ± 0.1	1.5 ± 0.1	46.6
	Ap4A	54 ± 16	2.6 ± 0.5	1.3 ± 0.3	47.4
S237A	UDPGlc	249 ± 8	26.2 ± 0.7	13.6 ± 0.4	105.1
	UDPGlcNAc	388 ± 50	19.6 ± 0.3	10.2 ± 0.1	50.5

All measurements were carried out at 25 °C in 50 mM TrisHCl, 25 mM NaCl, 0.1 mM ZnCl_2_, pH 9.5. Values were calculated using nonlinear regression according to the Michaelis–Menten equation and represent mean ± SD from fit parameters of three separate experiments.

#### AMP product binding mode

**Figure 2:**
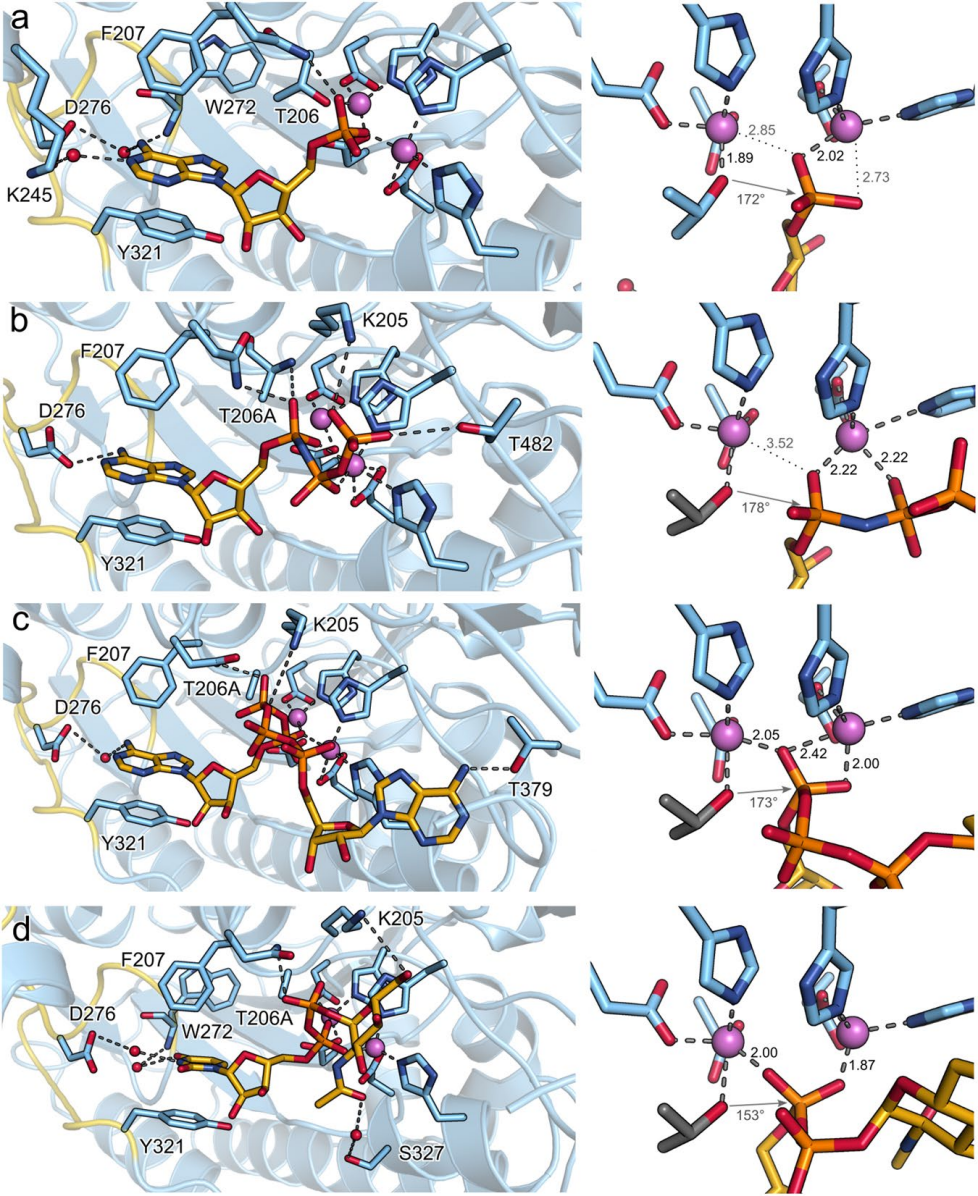
Substrate binding to NPP3. Crystal structures of (**a**) NPP3xAMP, (**b**) NPP3^T206A^xAMPNPP, (**c**) NPP3^T206A^xAp4A and (**d**) NPP3^T206A^xUDPGlcNAc. In the NPP3^T206A^ structures, T206 has been modeled based on the NPP3xAMP structures and is shown in grey. Water molecules and zinc ions are shown as red and purple spheres, respectively. No water molecules could be added for the AMPNPP complex structure due to the limited resolution. On the right side the coordination of the ligands to the dizinc site is depicted in a closer view. The angles between the T206 hydroxyl oxygen atom, the phosphorus atom, and the oxygen atom opposite to the T206 hydroxyl oxygen atom is specified. For the NPP3^T206A^xAp4A and NPP3^T206A^xUDPGlcNAc structures, the leaving group oxygen is not positioned opposite to the nucleophilic T206 hydroxyl group, indicating an unproductive binding mode.

**Figure 3:**
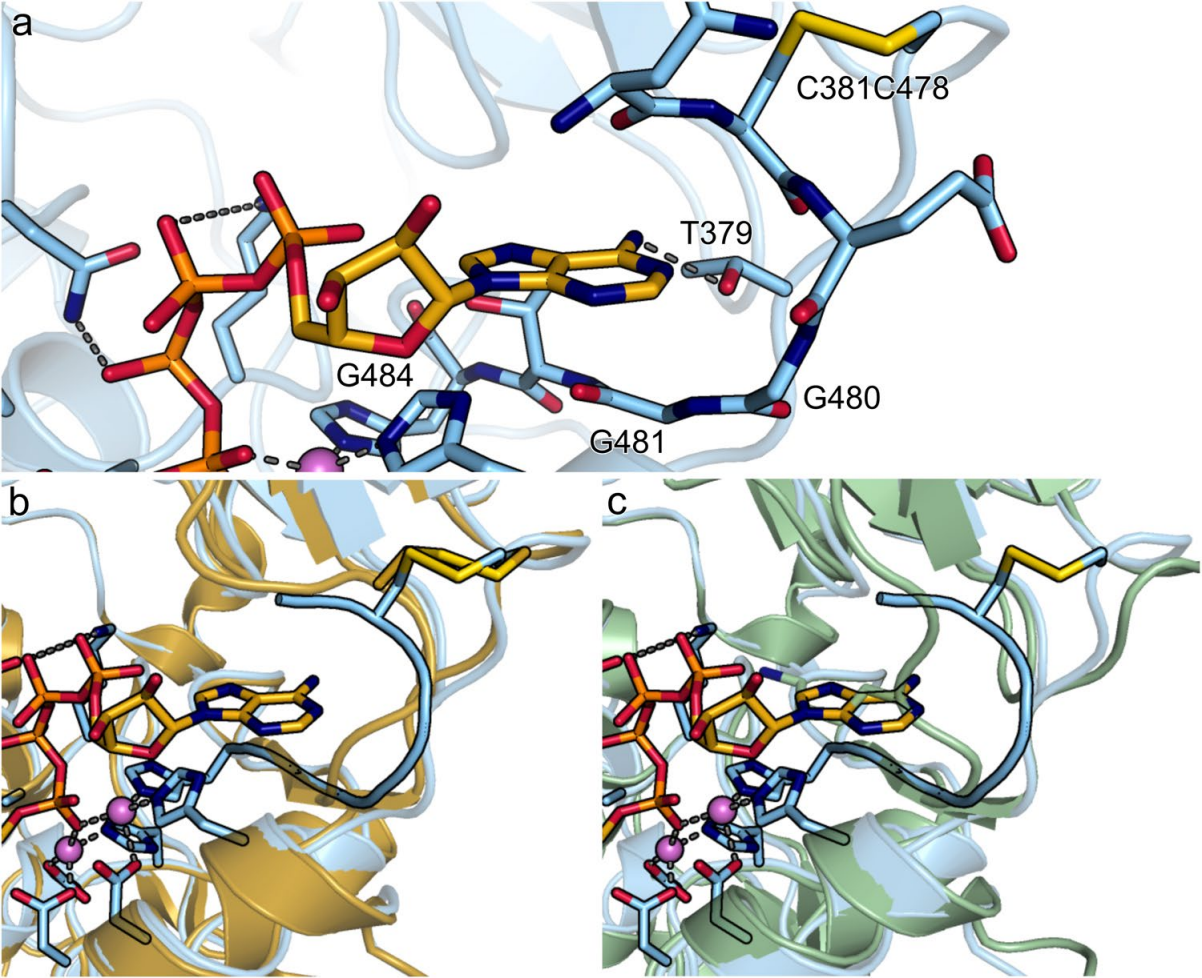
Comparison of the nucleotide binding pocket of (**a**) NPP3 (blue) with bound Ap4A to (**b**) NPP1 (4GTW) in yellow and (**c**) NPP4 (4LQY) in marine green. The structures have been superimposed based on the Cα atoms of the PDE domain.

Via cocrystallization we were able to bind the product AMP to NPP3 (Fig. 2a, Table 1). The nucleobase is sandwiched by π-π-stacking interactions between the aromatic residues F207 (edge to face) and Y290 (face to face). Polar interactions of the nitrogens of the adenine ring with the protein are formed via three water molecules (Figs 2a and 3a). The phosphate group of AMP is coordinated to Zn1 and hydrogen bonded to N227.

#### AMPNPP substrate binding mode

Despite the presence of structures of several NPP family members, a co-crystal structure with a substrate or substrate analog has not been described yet. After cocrystallization of NPP3 with 10 mM adenosine-5′-[(α,β)-methylene]triphosphate (AMPCPP), the electron density indicated no binding of the non-hydrolyzable substrate analog (data not shown). Cocrystallization of NPP3 with 10 mM adenosine-5′-[(α,β)-imido]triphosphate (AMPNPP) resulted in the observation of the hydrolyzed product AMP in the active site. We therefore created an inactive mutant of NPP3 by replacing the T206 nucleophile with alanine (NPP3^T206A^). Nevertheless, despite short soaking times at room temperature only the bound product AMP was visible. A transfer of the crystals to 4 °C and ligand soaking with concentrations of 10–20 mM for 20–30 min finally lead to the observation of AMPNPP coordinated to the active center. Despite the somewhat limited resolution of 2.9 Å for this structure, the inhibitor is well defined by the electron density map (Fig. S1). Absence of negative difference density at the positions of the β- and γ-phosphate and the marginally higher B-values of these two groups (average 76.3 Å^2^) compared to the α-phosphate (69.4 Å^2^) indicate that AMPNPP is bound with almost full occupancy and that there is no significant alternative binding of AMP.

The AMP moiety of the AMPNPP substrate analog generally has the same conformation and interactions compared to the AMP product binding mode (Fig. 2b). However, the nucleotide is positioned by ~0.7 Å closer to D276 such that the carboxylate group of this residue forms a direct hydrogen bond to the adenine amine group. Due to this shift, the α-phosphate group forms hydrogen bonds to the side chain of N227 and the backbone NH of T206 in addition to the Zn1-coordination. The β-phosphate group is also coordinated to Zn1 such that this zinc ion is five-coordinated. The terminal phosphate group may be bound by K205, which however has weak side chain density indicating flexibility. In close proximity for an additional hydrogen bond is T482. The mutations K205A and T482V reduce the specificity constant of NPP3 about six-fold and three-fold, respectively, mainly by reducing *k*_cat_ (Table 2). With an O-P-O angle of 178° the leaving group is almost perfectly positioned relative to the T206 nucleophile, which was modelled according to the AMP-bound structure. This indicates a catalytically productive binding mode.

### Recognition of dinucleotides

#### Diadenosine tetraphosphate (Ap4A) co-crystal structure

We determined a complex structure of NPP3^T206A^ with the dinucleotide Ap4A using the same protocol as described for AMPNPP (Fig. 2c). One adenine base of the compound is bound in a similar manner as observed before for bound AMP. Major differences are observed in the ribose conformation, which is 2′-*endo* in the Ap4A-bound structure, whereas it is 3′-*endo* in the AMP- and AMPNPP-bound structures, and in the coordination of the α-phosphate group. This phosphate is coordinated with one oxygen to Zn2 and the oxygen which would be opposite of the T206 nucleophile is coordinated to Zn1. In contrast to the AMPNPP-bound structure the β-phosphate group is not coordinated to Zn1 but is hydrogen-bonded to N227, which is thus not bound to the α-phosphate group. In this orientation, the β-phosphate group is not positioned opposite to the attacking nucleophile for an in-line phosphoryl transfer. It follows that the observed binding mode is catalytically non-productive. Nevertheless, we speculate that the binding mode of the secondary (“leaving group”) nucleoside moiety resembles that in a productive binding mode. It is located in a binding pocket formed by the loop region of residues N477 to G484, which includes three glycines (G480, G481 and G484; Fig. 3a). Human NPP1 and NPP3 have a conserved GXGXXG motif, which was previously noted as a consensus sequence for the dinucleotide binding enzymes NPP1 and NPP3^1^ based on the participation of glycine-rich sequences in nucleotide and dinucleotide binding sites in protein kinases^39^. In rat NPP3 this motif appears as GGXXG. Gly480 and Gly481 are in close contact to the adenine base, whereas Gly484 is not in near distance to make any interaction with the bound ligand. Mutation of all three glycines to alanine lead to an increased *K*_M_ value and a decreased turnover rate, but did not inhibit the catalytic activity completely (Table 2).

Additionally, the amine group of the adenine base of the leaving group forms a hydrogen bond with T379. This residue is highly conserved for NPP3, but not present in other members of the NPP family. A T379V mutation resulted in a more than 10-fold reduction of *k*_cat_/*K*_M_ for Ap3A and Ap4A (Table 2). A further component of the binding loop for the secondary nucleoside moiety is a disulfide bridge between C381 and C478. This cystine link is conserved for NPP1 and NPP3 and might fix the loop in an orientation for dinucleotide binding (Fig. 3b). NPP4 is lacking this disulfide bridge, which has the effect that the loop is folded in a different manner and therefore blocks the dinucleotide binding pocket via K334 (human NPP4 numbering). Therefore, the dinucleotide may bind in a more unfavorable way to NPP4 (Fig. 3c). This could explain the reduced catalytic efficiency of Ap3A hydrolysis by NPP4 (*k*_cat_/*K*_M_ = 11.7 M^−1^ s^−1^) compared to NPP1 (360.0 M^−1^ s^−1^) and NPP3 (639.9 M^−1^ s^−1^)^31^. NPP4 is known to stimulate platelet aggregation and secretion on the surface of brain vascular endothelium by hydrolyzing Ap3A^6^. The potent dinucleotidase activity shown in this study leads to the question whether NPP3 might have a similar function in hemostasis, albeit not having been described to be localized on vascular endothelium cells yet.

#### Binding of UDP sugars

NPP3 has been described as a regulator of glycosyltransferase activity by hydrolyzing UDPGlcNAc^19^. A complex structure of NPP3 with UDPGlcNAc showed that the recognition of the uracil group is similar to that of adenine (Fig. 2d). The 6-membered ring is stacked by F207 and Y290. Additionally the uracil base is oriented via two water mediated hydrogen bonds. D276 interacts with the N3 atom of the substrate via one water molecule and the backbone NH of W272 interacts with the C4 carbonyl group via another water molecule. Similar to the situation of the NPP3xAp4A co-crystal structure, the orientation of the α- and β-phosphate groups does not correspond to a catalytically productive binding mode for an inline phosphoryl transfer. The N-acetylglucosamine group is well defined in the electron density map and its atomic B-factors are by ~10 Å^2^ higher than those of the ribose group, indicating a slightly increased flexibility. The acetylated sugar forms no direct polar interactions with the protein but is packed against the T482 and H330 side chains. S237 is interacting by a water-mediated hydrogen bond with a hydroxyl group of the sugar. K205 forms van-der-Waals contacts to the sugar. The participation of T482, S237 and K205 in substrate binding could be verified by mutation experiments (Table 2).

## Discussion

### Specificity for the nucleobase, dinucleotides and UDP sugars, implications for other NPP family members

**Figure 4:**
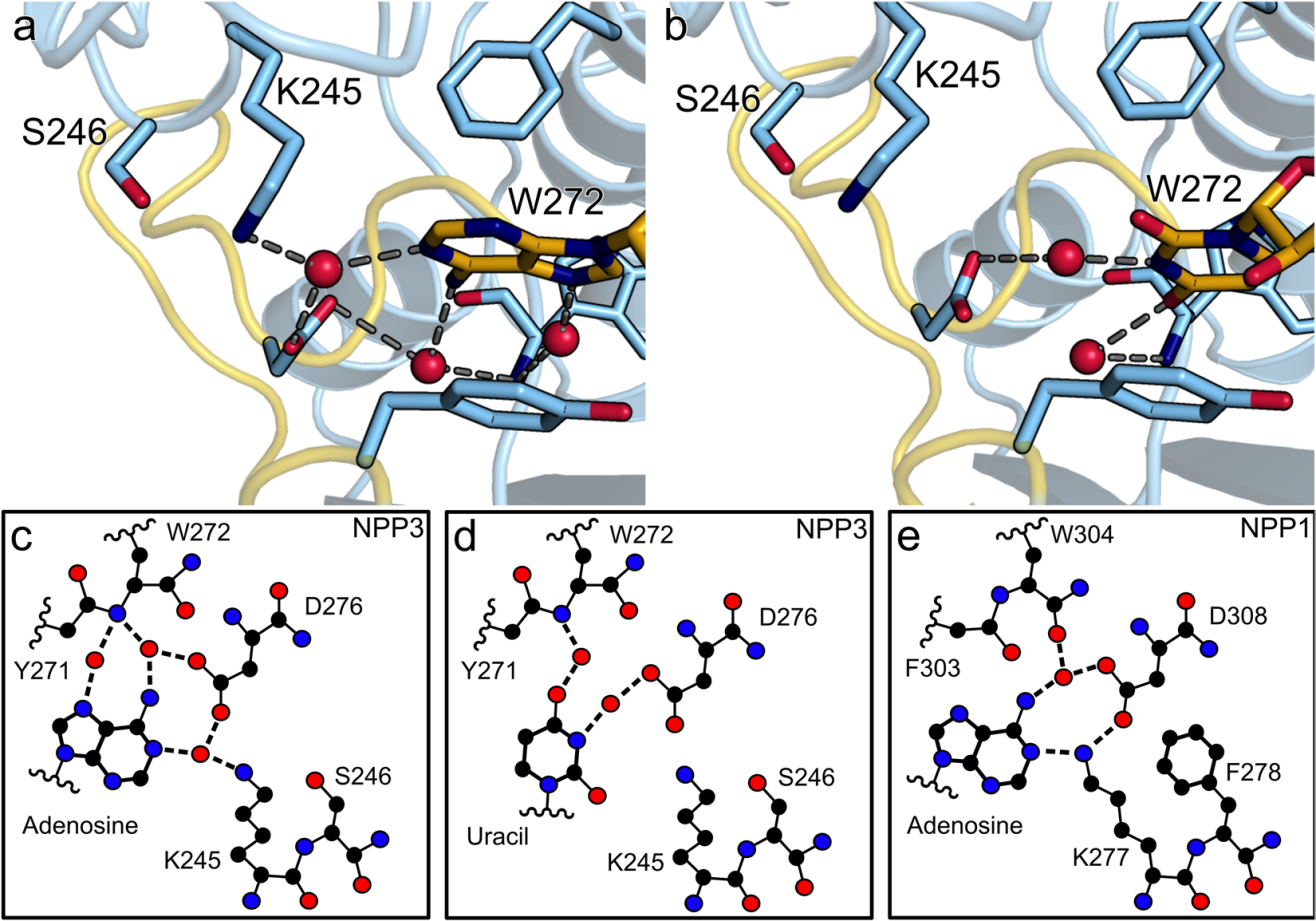
Nucleobase recognition. Water mediated interactions of NPP3 with the nucleobase of (**a**) AMP and (**b**) UDPGlcNAc. Schematic representation of the interactions between (**c**) NPP3 with AMP, (**d**) NPP3 with UDPGlcNAc and (**e**) NPP1 with AMP. Residues involved in nucleotide base recognition and nucleotides are shown by stick models. Black dashed lines indicate hydrogen bonds.

The kinetic parameters determined for four different nucleoside triphosphates showed no clear preference for one base type (Table 2). This is in clear contrast to the nucleotide preference of NPP1 which favors ATP over the other nucleotides^28,31^. NPP4 does not hydrolyze nucleoside triphosphates but is specific for Ap3A and Ap4A^31^. The ATP preference of NPP1 was explained with hydrogen-bonding interactions between a water molecule, N6 of AMP, the main chain carbonyl group of W304 and the side chain of D308, which recognize specifically the adenine base^28^. In NPP3 however, a similar hydrogen-bonding network is observed in the NPP3xAMP co-crystal structure. A main difference between the NPP3xAMP and NPP1xAMP complex structures is the direct coordination of K277 of NPP1 to N1 of the adenine base. In NPP3, the corresponding K245 does not directly interact with the adenine base, but does so via a water molecule. This is possible due to the absence of F278 of NPP1, which is replaced by the polar and less bulky side chain of S246 in NPP3. This enables a second water molecule to bind between D276 and the adenine ring at about the same position as the K277 amine group in NPP1 (Fig. 4). In the NPP1 structure, F278 appears to fix K277 in a conformation for this direct interaction with the adenine N1. NPP2 and NPP4 possess at this position an equally bulky amino acid as NPP1 suggesting a similar base recognition. However, the nucleobase preference has only been characterized for NPP1, NPP3 and NPP4^28,31^. Smaller residues at this position can be found for NPP6, NPP7 and *Xa*NPP.

The more flexible base recognition via water molecules may be required for NPP3 for its suggested function in the hydrolysis of UDPGlnNAc in the ER and Golgi. In Neuro2s cells NPP3 may serve as an inhibitory regulator of the N-acetylglucosaminyltransferase GnT-IX by hydrolyzing nucleotide sugars^19^.

Similar to the situation in NPP1 a “WPG loop” of 19 amino acids (residues 272–290) within the catalytic domain of NPP3 prevents the formation of a hydrophobic pocket for binding lysophospholipids as seen in NPP2^30,40^. Thus, NPP1 and NPP3 lack lysophospholipid hydrolyzing activity^41^.

NPP3 does also efficiently hydrolyze dinucleotides (Table 2). The hydrolytic efficiency of rat NPP3 towards Ap3A and Ap4A is comparable with human NPP1 (Ap3A: *k*_cat_ = 7.2 s^−1^, *K*_M_ = 20.0 μM)^31^. NPP3 hydrolyzes Ap3A and Ap4A slightly more efficiently than nucleoside triphosphates whereas the specificity for UDPGlc, UDPGlcNAc and NAD^+^ is comparable. These data suggest that recognition of these substrates is mainly based on binding of the nucleoside diphosphate moiety common to all substrates. This finding is in agreement with the results from the crystal structure analyses of Ap4A and UDPGlcNAc complexed to NPP3, which show less interactions with the protein and higher B-factors of the leaving group moieties.

While NPP1 and NPP3 exhibit good activity towards ATP and adenine dinucleotides, NPP4 hydrolyzes the dinucleotides Ap3A and Ap4A efficiently, but cleaves ATP much slower^31^. In a comparison of the active sites of NPP1 and NPP4 it has been suggested, that charge stabilization of the ATP γ-phosphate by a lysine clamp consisting of three lysine residues might be responsible for the much higher ATP activity of NPP1 (*K*_M_ = 144.5 µM and *k*_cat_ = 7.8 s^−1^) compared to NPP4, which has two lysines positioned for coordination of the γ-phosphate^31^. NPP3 has similar kinetic parameters for ATP hydrolysis (*K*_M_ = 44.1 µM and *k*_cat_ = 10.0 s^−1^) as NPP1, but only one positively charged residue (K205) in vicinity of the γ-phosphate. A K205A mutation in NPP3 increases *K*_M_ slightly and decreases the turnover rate (*K*_M_ = 53.8 μM and *k*_cat_ = 2.0 s^−1^), however, ATP hydrolysis is quite efficient even though no lysine residue is positioned for binding the terminal phosphate group. These findings argue against the lysines alone influencing ATP specificity. A further difference in NPP4 is D335, which is a threonine in NPP3 and a phenylalanine in NPP1, as suggested before^31^. D335 would be in hydrogen-binding distance to the γ-phosphate group of AMPNPP, if it were bound as in NPP3. D335 thus might prohibit a productive ATP binding mode in NPP4 via charge repulsion.

### NPP catalytic mechanism

In addition to the characterization of the NPP3 structure and substrate specificity of the enzyme, the determination of a co-crystal structure with a substrate analog (AMPNPP) is a main result of the present study. Information on the catalytic mechanism of NPPs was first obtained based on sequence comparisons and mutational data demonstrating that the NPP catalytic center closely resembles that of the enzyme alkaline phosphatase (AP)^42^. It was suggested, that the substrate binding modes in the two-step mechanism match those of the suggested AP mechanism, which is well studied by crystallographic and other data (Fig. S2)^3,43,44^. Mutational and structural work on NPPs later supported this basic catalytic scheme and X-ray as well as EXAFS data on *E. coli* AP and *Xanthomonas citri* NPP (*Xac*NPP) demonstrated similar binding modes of substrates and the vanadate transition state analog^45^. However, the AMPNPP binding mode observed with NPP3 differs in details of the coordination of the substrate phosphate to the metal ion from the prototypical AP mechanism, in which the substrate is suggested to bind with one free oxygen atom to the Zn2 ion where also the nucleophile is coordinated, whereas the leaving group oxygen coordinates to Zn1 (Fig. S2)^46^. In the AMPNPP binding mode of NPP3, one of the free oxygen atoms is bound to Zn1 and the leaving group oxygen is not metal-coordinated (Figs S2, S3). In the NPP3xphosphate and NPP3xAMP co-crystal structures a similar binding mode of the phosphate group is observed.

A comparison of available structures of NPP substrate binding modes shows that there is a significant variation in the exact binding mode of the zinc-coordinated phosphate group (Figs S3, S4). A binding mode corresponding to the prototypical AP Michaelis complex depicted in Fig. S2 has so far only been observed for mouse NPP5 in complex with AMP^12^ and for mouse NPP6 in complex with phosphocholine (Fig. S4)^13^. A variation of this reaction scheme is seen for *Xac*NPP in complex with AMP (PDB id 2RH6), where the leaving group oxygen is also Zn1-coordinated but the free phosphate oxygen interacts with both zinc ions at rather long distances of 2.31 and 2.50 Å (Fig. S4)^47^. In all other binding modes the leaving group oxygen does not form a normal coordination bond to Zn1. The coordination bond length between a phosphate monoester and a 4-coordinated zinc ion should be around 1.97 Å^45^. The binding mode observed for the free phosphate ion, the AMP product complex and the AMPNPP substrate analog bound to NPP3 in this study has also been found in the product complexes of mouse NPP2 (3NKN, 3NKQ)^30^ and of human NPP4 (4LQY)^6^. A bridging coordination of the free phosphate oxygen to both zinc ions is only observed in AMP-bound mouse NPP1 (4GTW)^28^. In many other structures this oxygen atom is also oriented between the two zinc ions, but at rather long distances and often closer towards the Zn1 ion (*rn*NPP2: 5DLW^48^; *hs*NPP2: 4ZG7^49^; *mm*NPP2: 3NKP^30^).

The fact that such differences in the metal-coordination of the phosphate group are also observed for the same or closely related enzymes indicates that these probably do not reflect mechanistic variations between different NPP family enzymes alone. Obviously, almost isoenergetic binding modes for the phosphate group exist. Differences in details of the active site structure, pH value, substrate structure, and enzyme conformation influenced by crystal packing effects (corresponding to likely thermally accessible conformations in solution) might change the relative energy of these binding modes. From the crystal structures alone, we cannot tell if all these binding modes are indeed catalytically productive. All binding modes shown in Figs S3 and S4 appear to be productive based on the orientation of the threonine nucleophile, the phosphorus atom and the leaving group oxygen positioned for an in-line phosphoryl transfer.

Further information on the catalytic mechanism and the relevance of the observed phosphate binding modes on catalysis might be obtained by transition state analogs. Co-crystal structures with orthovanadate forming a five-coordinate trigonal bipyramidal transition state have been obtained for rat NPP2 (5IJS)^50^ and *Xac*NPP (2GSO)^47^. The VO_5_ binding mode observed for rat NPP2 supports a mechanism in which the free oxygen is coordinated only to Zn1 and the leaving group oxygen is not metal-coordinated (Fig. S4). This transition state binding mode corresponds best to the substrate binding mode observed for the NPP3 structures in this study. In contrast, in the *Xac*NPPxvanadate complex structure, the leaving group oxygen is coordinated to Zn1 due to the distortion of the VO_5_ coordination sphere from a linear trigonal bipyramidal structure (Fig. S4).

Coordination of the leaving group oxygen to Zn1 might promote catalysis by stabilization of a very basic leaving group. For the hydrolysis of phosphoanhydrate bonds as in nucleoside triphosphate cleavage, stabilization of the leaving group oxygen is not necessary as the diphosphate leaving group is stable in the deprotonated state at neutral pH. For the hydrolysis of the phosphoester bond in phospholipids by NPP2, however, Zn1-coordination of the basic alkoxide leaving group might be necessary for efficient hydrolysis. Alternatively, the leaving group is protonated by a water molecule hydrogen bonded to D473 in rat NPP2.

**Figure 5:**
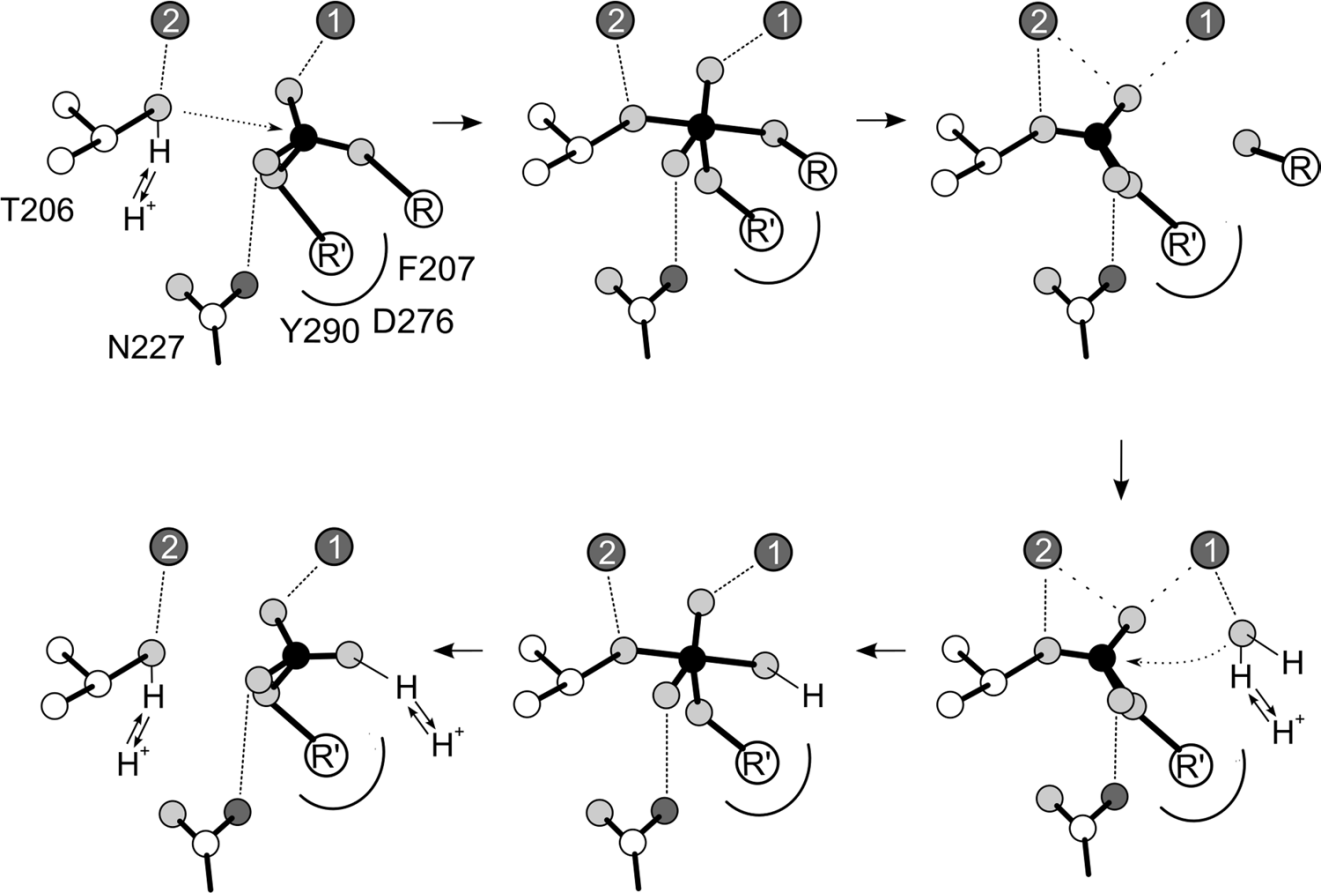
Suggested catalytic mechanism of NPP3. This mechanism is mostly based on the NPP3 substrate and product complex structures reported in this work and on the rat NPP2 x VO5 transition state analogue complex structure^50^. See text for further details.

Based on these structures and considerations, we propose a hydrolytic mechanism for NPP3, in which the leaving group oxygen is not metal-coordinated (Fig. 5). In the first phosphoryl-transfer step, Zn1 coordinates the phosphate group and participates in transition-state stabilization while Zn2-coordination facilitates deprotonation of the threonine nucleophile. In the second phosphoryl transfer reaction, the hydrolysis of the phosphorylated intermediate, we assume that the water nucleophile is coordinated to Zn1 for deprotonation to a hydroxide ion. The binding mode of the transition state is suggested to match that of the forward reaction. This is based on the assumption that the strong stabilization of a transition state necessary for efficient catalysis is achieved by an evolutionary optimized active site geometry with a single binding mode and very specific interactions with the transition state. It thus appears unlikely that the two similar and mostly symmetric phosphoryl transfer steps of the NPP catalytic mechanism are catalyzed via different interactions of the enzyme and transition state structures.

In summary, the NPP3-substrate structures analyzed in this study reveal details about substrate specificity and recognition, which is in agreement with the kinetic and mutational analysis of the enzyme. A comparison with other NPP structures indicates that the AP-like catalytic mechanism of NPP enzymes involves almost isoenergetic substrate binding modes, which differ in the coordination of the free oxygen atom and the leaving group oxygen atom to the two metal ions. It remains unclear from the crystal structures alone, if the phosphoryl transfer reaction can only proceed via one of these substrate binding modes or if the transition state can be reached from several binding modes. Quantum chemistry calculations may help to further analyze this question based on the detailed structural information now available from several crystal structures of NPP enzymes.

## Methods

### Protein preparation

For kinetic and structural investigation, we expressed rat NPP3 transiently in HEK293S GntI^−^ cells^51^. In these mutant HEK cells the lack of N-acetyl-glucosaminyltransferase leads to a homogenous glycosylation pattern, which increases the chance of crystallization. The used construct contains the complete catalytic and nuclease-like domains (residues 140–875), but lack the SMB-like domain. Proteins were prepared as described previously (Doehler *et al*., Acta Cryst F., under review). Briefly, residues 140–875 were fused with an N-terminal secretion signal provided by the expression vector pHLsec^52^ and a C-terminal His_6_-tag for affinity purification. The protein was expressed transiently with PEI as transfection reagent. After buffer exchange the secreted protein was purified from the HEK cell culture medium by immobilized metal ion affinity chromatography using a HisTrap HP column (GE Healthcare) and further with size exclusion chromatography on a Superdex 200 16/60 gel filtration column (GE Healthcare). The NPP3 mutants were constructed by site directed mutagenesis and prepared using the same protocol as for the wild type. The mutagenesis primers are listed in Table S2.

For determination of the kinetic data, the complete ectodomain of NPP3 (residues 49–875) was expressed in HEK293T cells and purified as described previously (Doehler *et al*., Acta Cryst F., under review).

### Crystallization, data collection and structure determination

The protein was crystallized as described previously (Doehler *et al*., Acta Cryst F., under review). In summary, for crystallization the hanging-drop vapor-diffusion method was used at 19 °C. Drops were produced by mixing 1 µL of protein solution (5 mg/ml in 20 mM Tris-HCl, pH 8.0) and 1 µL of reservoir solution (0.2 M KCl, 0.1 M magnesium acetate, 0.05 sodium cacodylate pH 6.0, 10.2% (*w*/*v*) PEG8000, 1 mM CaCl_2_, 0.1 mM ZnSO_4_). For the cocrystallization with AMP the protein was incubated with 10 mM of AMP directly before the preparation of the crystallization mixture. For soaking experiments, crystals were grown at 19 °C and stored at 4 °C overnight for thermal equilibration of the crystallization plates and buffers. Crystals were then transferred to a drop containing the reservoir buffer and the respective ligand at a concentration of 5 to 20 mM for 10–20 min. For cryoprotection glycerol was added stepwise to a concentration of 12% (*v*/*v*) over 5 min and the crystals were directly flash frozen in liquid nitrogen. X-ray data were collected at beamline 14.1 of the Helmholtz Center Berlin (Germany) using a Pilatus6M detector. Data reduction was performed by XDS^53^. The structure of NPP3 was solved by molecular replacement with the program PHASER^54^, using a NPP3 model based on the crystal structure of mouse NPP2^30^. The molecular replacement search was followed by rigid-body refinement with REFMAC5^55^. The model was improved with manually building in COOT^56^ and refined with BUSTER^57^. The model quality was validated by the wwPDB validation tool^58^.

### Measurement of enzymatic activity using isothermal titration calorimetry (ITC)

We used the multiple-injection method with a stepwise addition of substrate to enzyme. This method prevents the problem of product inhibition, which occurs for NPP3 by the single-injection method due to high product and low substrate concentrations at the end of substrate hydrolysis. Prior to the determination of catalytic constants the molar reaction enthalpy of each nucleotide was measured using an optimized reaction buffer (Table S1). For optimization of the reaction buffer regarding pH, salt concentration and Zn^2+^ concentration a colorimetric reaction assay by measuring the quantity of p-nitrophenolate at 405 nm released from the artificial substrate p-nitrophenyl thymidine-5′-monophosphate (pNP-TMP) was used.

**Figure 6:**
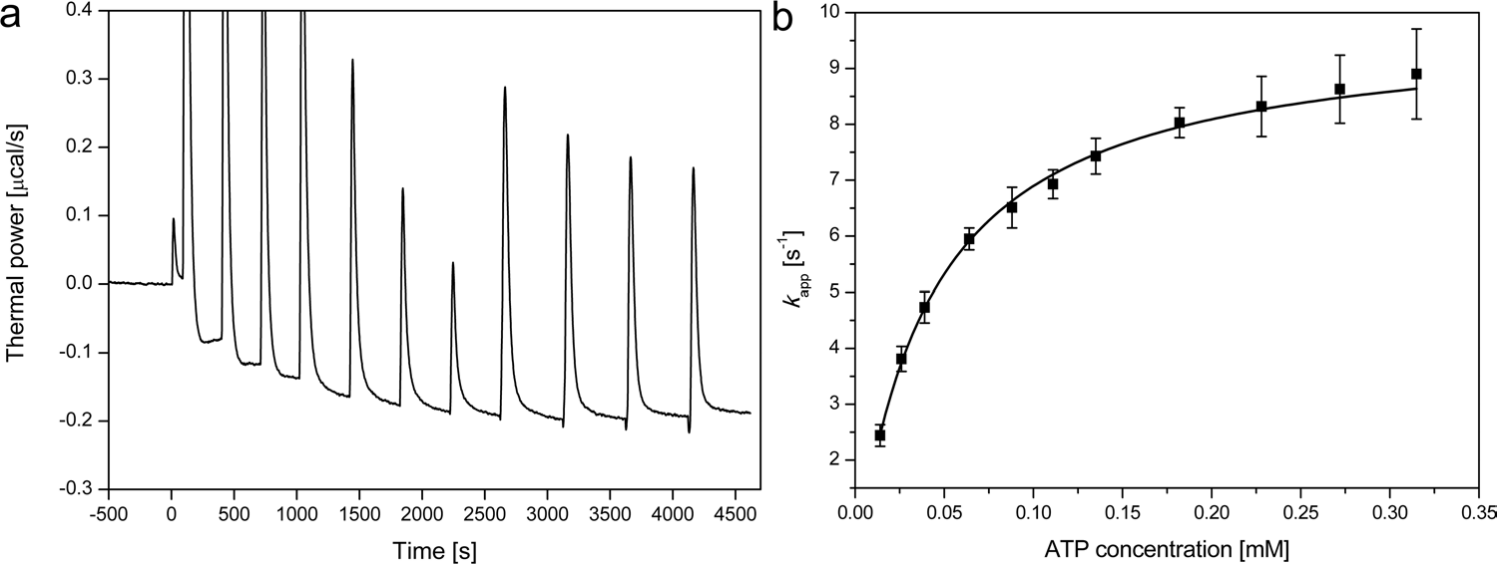
Determination of kinetic parameters by ITC. (**a**) Titration experiment with 12 injections (1 × 0.5 µL, 3 × 10 µL, 4 × 20 µL and 4 × 40 µL) of 2 mM ATP to 1.32 µM NPP3 (E49-I815) with reequilibration times of 310 to 500 s. (**b**) Plot of the derived turnover rates against the substrate concentration and fit of Michaelis-Menten equation. The error bars are derived from three independent experiments.

Catalytic parameters for NPP3 were determined using a MicroCal (Freiburg, Germany) VP-ITC, which directly measures heat evolved in liquid samples, in this case during hydrolysis of the energy rich phosphoanhydride bonds of the nucleotides^38^. The reaction buffer contained 50 mM TrisHCl, 25 mM NaCl, 0.1 mM ZnCl_2_ at pH 9.5 and was prepared from a 2x concentrated stock solution. For each experiment the sample cell was filled with 1500 µL protein solution with a protein concentration between 0.5 and 5 µM depending on the turnover rate for the substrate. The injection needle, which had a capacity of 280 µL, was stirred at 307 rpm. Nucleotides provided in the syringe at a concentration between 1 and 5 mM according to the binding affinity were injected within 20 s. After each injection a reequilibration time of 310–500 s was required to compensate dilution heats. The recorded thermal power data were converted to turnover rates using the molar reaction enthalpy and further with the enzyme concentration to apparent rate constants using the data-analyzing software Origin Pro 8 G (OriginLab). These apparent rates could be fitted to the Michaelis-Menten equation to derive the kinetic parameters *K*_M_ and *k*_cat_ from three separate experiments. A typical experiment consisted of 1 × 0.5 µL, 3 × 10 µL, 4 × 20 µL and 4 × 40 µL injections of substrate to enzyme at 25 °C (Fig. 6).

The molar enthalpy change Δ*H*_r_ of nucleotide hydrolysis was determined by injecting 90 µL of a 2 mM solution of nucleotide into the reaction buffer without enzyme to obtain the dilution heat (Δ*H*_d_). In the presence of a suitable enzyme concentration the total heat (Δ*H*_t_) after complete hydrolysis was obtained. Subtraction of Δ*H*_d_ from Δ*H*_t_ yields Δ*H*_r_. For Ap4A, which is hydrolysed via ATP to AMP, Δ*H*_r_ of the first hydrolysis step was determined by subtraction of Δ*H*_r_ for ATP from Δ*H*_r_ obtained for hydrolysis of Ap4A to AMP.

### Data availability

Protein Data Bank: The atomic coordinates and structure factors for the reported crystal structures are deposited under accession codes specified in Table 1. Additional data are available on request from the corresponding author (N.S.).

## Electronic supplementary material

Supplementary information
