# Supplementary material for: Crystal structure and substrate binding mode of ectonucleotide phosphodiesterase/pyrophosphatase-3 (NPP3)
Source: Sci Rep. 2018 Jul 18;8:10874. doi: 10.1038/s41598-018-28814-y (PMC6052110; doi:10.1038/s41598-018-28814-y)
Supplement: Supplementary file 1 — Supplementary information [file 41598_2018_28814_MOESM1_ESM.docx]

**Supplementary Material**

*Crystal structure and substrate binding mode of ectonucleotide phosphodiesterase/pyrophosphatase-3 (NPP3)*

Christoph Döhler, Matthias Zebisch, Norbert Sträter


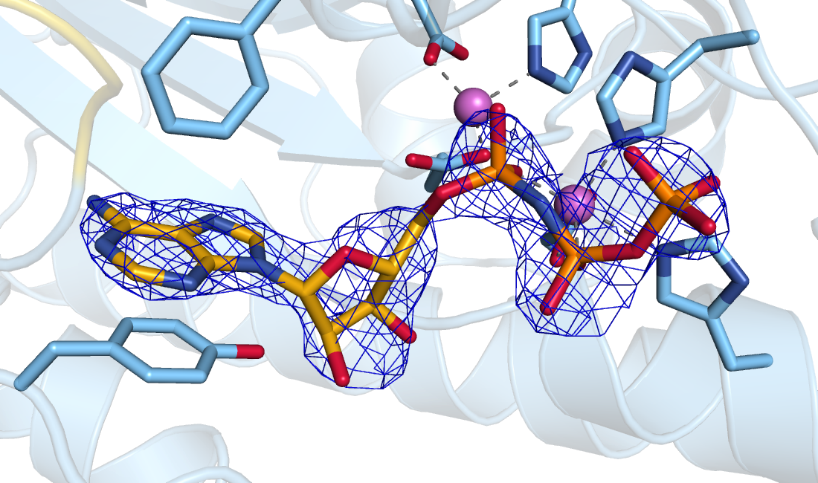


**Supplementary Figure S1**: Electron density map of AMPNPP bound to the active site of NPP3. The feature enhanced map (2m*F*_o_-*F*_c_) is contoured at 2.2 σ.


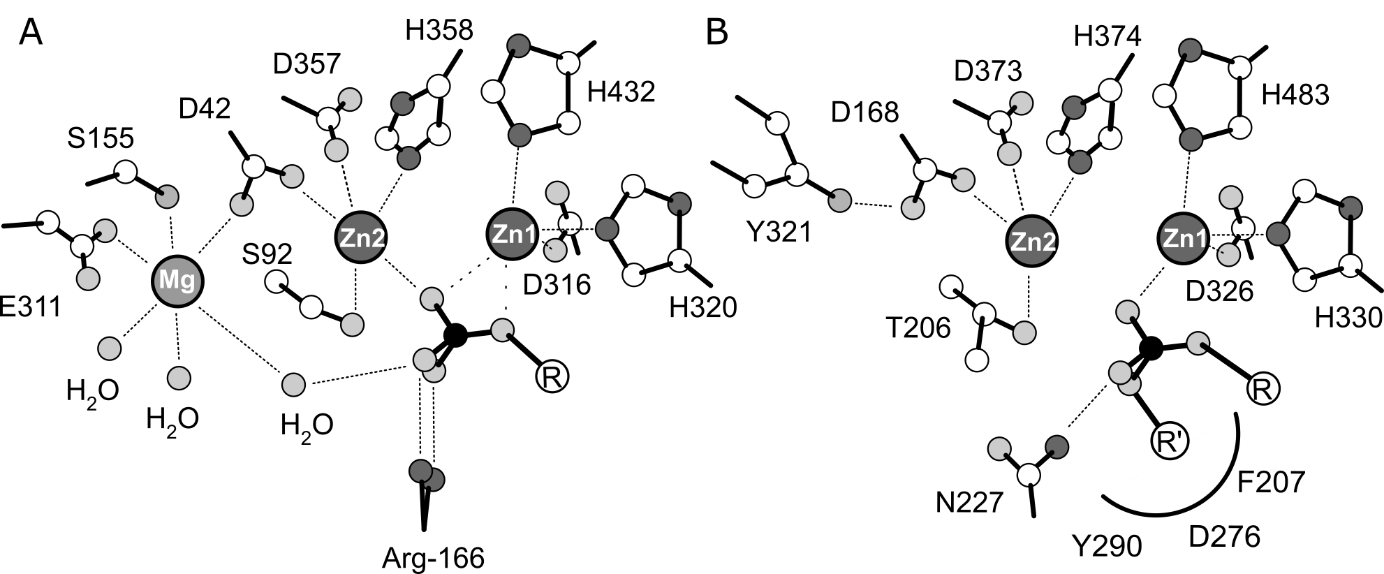


**Supplementary** **Figure S2**: Comparison of substrate binding modes and active site structure of human alkaline phosphatase (a) and rat NPP3 (b).


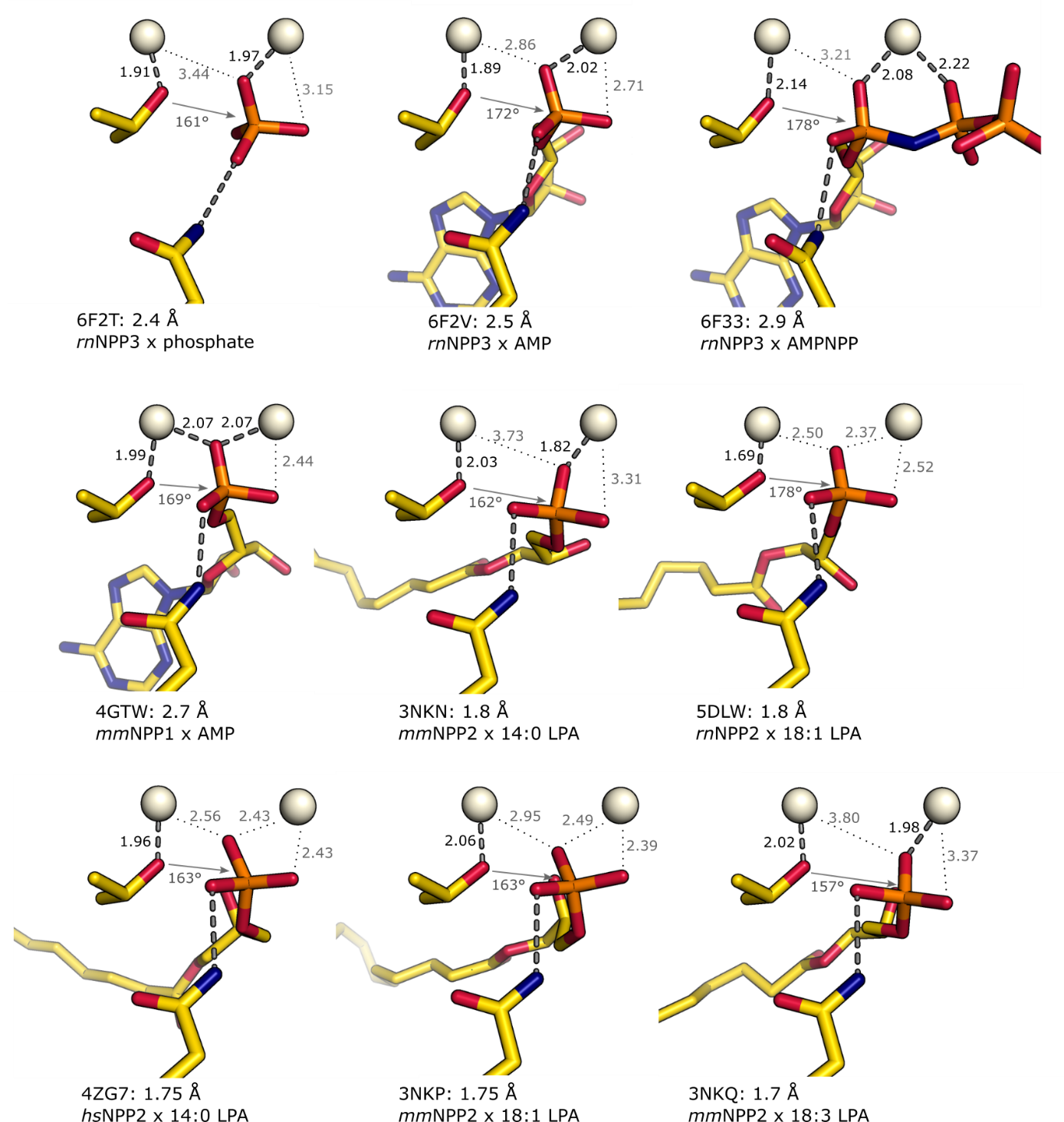


**Supplementary** **Figure S3**: Comparison of binding modes of the phosphate groups in various product complex structures to the dizinc center of NPP1-3.


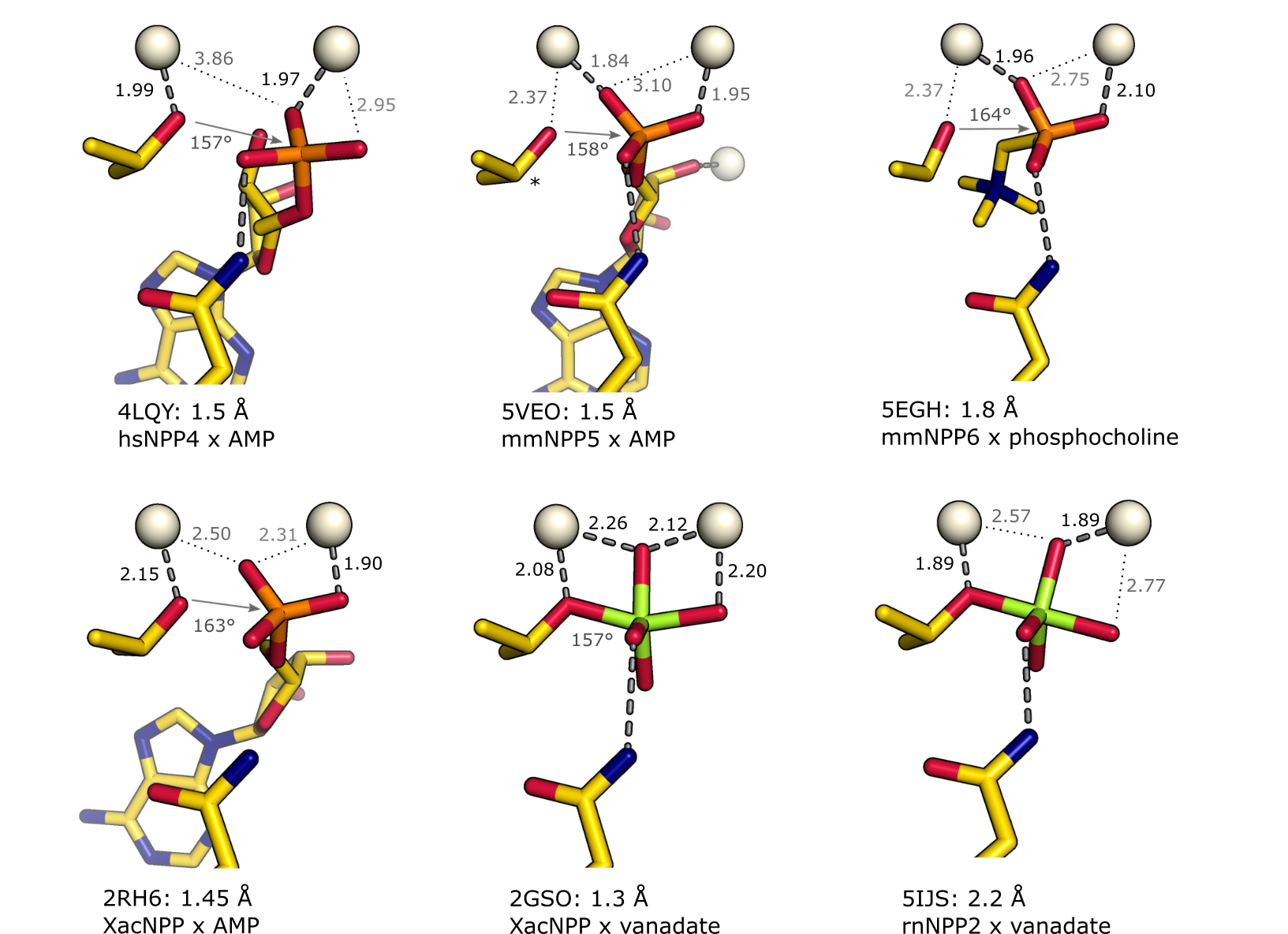


**Supplementary** **Figure S4**: Comparison of the binding mode of phosphate groups in various product complex structures and vanadate to the dizinc center of NPP4-6. In the structure of mmNPP5 (pdb id 5VEO) the Thr72 nucleophile (*) is modeled in a low energy conformer for the generation of this figure. For structure analysis, a T72A mutant was used. The structure pdb id 2RH6 of XacNPP is a higher resolution structure of this complex analyzed by the same authors earlier at 2.0 Å as PDB id 2GSU (Zalatan et al. 2006). 2RH6 differs in the phosphate group coordination from 2GSU, in which the AMP has only 60 % occupancy in monomer A and close contacts in monomer B of the asymmetric unit. 2RH6 appears to have no associated publication. The cocrystal structure of *hs*NPP7 in complex with phosphocholine is not included here because the phosphate group is not in a productive binding mode for phosphoryl transfer in this complex.

**Supplementary** **Table S1**: Molar reaction enthalpies of nucleotide hydrolysis in 50 mM Tris/HCl, 25 mM NaCl, 0.1 mM ZnCl_2_, (pH 9.5).

| Reaction | Δ*H*_t_ [kcal/mol] | Δ*H*_d_ [kcal/mol] | Δ*H*_r_ [kcal/mol] |
| --- | --- | --- | --- |
| ATP → AMP + PPi | -16.22 ± 0.74 | 0.66 ± 0.08 | -15.66 |
| UTP → AMP + PPi | -17.17 ± 1.52 | 0.71 ± 0.09 | -16.45 |
| GTP → AMP + PPi | -22.41 ± 3.47 | 0.16 ± 0.02 | -22.26 |
| CTP → AMP + PPi | -19.85 ± 0.89 | 1.11 ± 0.02 | -18.74 |
| Ap3A → AMP + ADP | -26.73 ± 0.01 | 0.50 ± 0.08 | -26.23 |
| Ap4A → AMP + ATP | -38.66 ± 0.32 | 0.29 ± 0.14 | -38.36 |
| Ap4A → 2 AMP + PPi | — | — | -22.70 |
| UDPGlc → UMP + PGlc | -29.89 ± 2.26 | -0.05 ± 0.01 | -29.53 |
| UDPGlcNAc → UMP + PGlncNAc | -36.22 ± 0.42 | -0.03 ± 0.01 | -36.25 |
| NAD^+^ → AMP + NMP | -27.38 ± 0.44 | 0.14 ± 0.05 | -27.24 |

**Supplementary** **Table S2**: Overview of the primers for the generation of NPP3 mutants

| Mutation site | Primers |
| --- | --- |
| K205A | 5’-GAGAGCAGTGTATCCCACC**GCA**ACCTTTCCAAATCATTATACC-3’  3’-CTCTCGTCACATAGGGTGG**CGT**TGGAAAGGTTTAGTAATATGG-5’ |
| S237A | 5’-CCGAT**GCT**GCGGGGCACAAGAGTGGA-3’  3’-ATACACCTTCTCGGGCGG**CTA**C-3’ |
| T379V | 5’-CCAG**GTC**TCTTGTGACAGAGTGGAATAC-3’  3’-CTAGTACCTTACCTGGTC**CAG**AGAAC |
| G480A, G481A, G484A | 5’-GGGAGTTCCAACTGCGAA**GCCGCC**ACACAC**GCT**TACAACAATGAATTTAAAAG-3’  3’-CCCTCAAGGTTGACGCTT**CGGCGG**TGTGTG**CGA**ATGTTGTTACTTAAATTTTC-5’ |
| T482V | 5’-CGGGGT**ACA**CGGTTACAACAATGAA-3’  3’-AAGGTTGACGCTTCCGCCCCA**TGT**GCC-5’ |
